# Elucidation of “Twig Canker and Shoot Blight” (TCSB) in Peach Caused by *Diaporthe amygdali* in the North of Italy in Emilia‐Romagna

**DOI:** 10.1111/ppl.70428

**Published:** 2025-07-31

**Authors:** Federico Brugneti, Luca Rossini, Marco Cirilli, Alessia Nardecchia, Mattia Onofri, Angelo Mazzaglia, Silvia Turco

**Affiliations:** ^1^ Dipartimento di Scienze Agrarie e Forestali, Università Degli Studi Della Tuscia Viterbo Italy; ^2^ Service D'automatique et D'analyse Des Systèmes Université Libre de Bruxelles Brussels Belgium; ^3^ Dipartimento di Scienze Agrarie ed Ambientali, Università Degli Studi di Milano Milano Italy; ^4^ Responsabile Frutta, Ufficio Produzioni e Servizi Agricoli, Conserve Italia Bologna Italy

**Keywords:** *Diaporthe amygdali*, molecular characterization, plant pathogens, plant pathology

## Abstract

*Prunus persica* (peach) is a major fruit species in global agriculture, with Spain and Italy being the leading producers in the European Union. As agriculture is subject to increasing pressure to reduce agrochemicals, effective management of diseases such as twig canker and shoot blight (TCSB) becomes crucial. *Diaporthe amygdali*, the primary fungal pathogen responsible for TCSB, causes a significant dieback of shoots, flowers, leaves, and branches, typically during late winter or early spring. Despite its considerable impact, there is a lack of comprehensive knowledge on the biological characteristics of this pathogen in Italian peach orchards. This study aimed to address these gaps by characterizing *D. amygdali* isolates from symptomatic trees in a productive region of northern Italy, Emilia Romagna. Morphological and molecular analyses were conducted, and mycelial extension was assessed at different temperatures to assess optimal growth conditions. The findings provide valuable insights into the thermal responses of *D. amygdali*, contributing to the development of decision support systems for more precise and targeted disease control strategies.

## 1 Introduction

*Prunus persica* (L.) Batsch, commonly known as peach, is a globally significant fruit crop within the Rosaceae family. Native to China, it has been extensively introduced and cultivated in many areas worldwide. Appreciated for its organoleptic properties and nutritional value, peach ranks among the most widely cultivated fruit crops worldwide, ranking second only to apple in terms of global production (Manganaris et al. 2022). Current data indicate China as the leading producer, with an average annual output of 17.5 million tons, followed by the European Union at 3.2 million tons (FAOSTAT 2023). Within the EU, Spain and Italy are prominent producers, with overall 1.2 million tons and 0.7 million tons in 2023, respectively (ISTAT 2024).

Given its widespread cultivation, *P. persica* is exposed to a range of biotic stressors that negatively impact yield, fruit quality, and plant longevity, thereby increasing production costs. Accordingly, effective plant protection practices are crucial for ensuring high‐quality production while minimizing traditional pesticide application. Notably, the re‐emergence of pathogens associated with Twig Canker and Shoot Blight (TCSB) has been observed in Italian peach orchards, particularly in Emilia‐Romagna (Agronotizie 2021). TCSB symptoms include rapid desiccation of shoots, flowers, leaves, and branches, typically occurring in late winter or early spring. Infected plants also show exudate leakage from cankers throughout the growing season, potentially attributed to fungal toxins, such as Fusicoccin A (Hilário et al. 2021, 2022).

*Diaporthe amygdali* is frequently identified as the primary fungal pathogen associated with (TCSB) in peach ( *P. persica* ; Hilário et al. 2021, 2022; Wang et al. 2021). However, other fungi, including *Botryosphaeria* spp., *Leucostoma persoonii*, *Cytospora* spp., and other *Diaporthe* species such as *D. eres*, have also been linked to the disease complex (León et al. 2020). As a consequence, further studies are needed to better characterize the etiological agent of TCSB and clarify the role of *D. amygdali* in the disease. Beyond Europe (Delacroix 1905; Canonaco 1936; Thomidis and Michailides 2009) and China (Dai et al. 2012), *D. amygdali* has been documented as a significant economic concern in both the United States (Farr et al. 1999) and Uruguay (Sessa et al. 2017). Initially described as *Fusicoccum amygdali* in France over a century ago (Delacroix 1905), the species was later reclassified into the *Diaporthe* genus based on multi‐locus phylogenetic analyses. These analyses involved several genetic markers, including the internal transcribed spacer (*ITS*) region of nuclear rDNA, translation elongation factor 1‐α (*TEF‐1*), β‐tubulin (*TUB2*), and calmodulin genes (*CAL*; Udayanga et al. 2012).

A comprehensive understanding of the biological and ecological characteristics of *D. amygdali* is crucial for either breeding programs or effective disease management. However, the current literature on this pathogen is still limited. To fill this gap in knowledge, a survey was conducted in symptomatic peach orchards within Emilia‐Romagna, employing culture‐based isolation, morphological and molecular characterization, and temperature‐controlled mycelial extension assays to identify virulent isolates. These findings will provide critical insights for developing targeted strategies to mitigate TCSB, thereby enhancing peach production sustainability and orchard resilience.

## 2 Materials and Methods

### 2.1 Field Survey and Fungal Isolation

In April 2024, a field survey was conducted in two peach orchards located in Emilia‐Romagna, Italy at 44°20′57.0″ N, 12°12′10.1″ E and 44°22′51.4″ N, 11°48′24.7″ E, to investigate the presence of twig canker and dieback symptoms. A total of 55 symptomatic twig samples were collected from the cultivars “Pavoro—Pav 2708,” “Pavoro—Pav 1705,” “Pavoro—Pav 2408,” “Pavoro—Pav 1505,” “Pavoro—Pav 2308,” “Fergold,” ‘Redhaven,’ “Glohaven,” “Lami.COM,” and “Lami.IT.” The samples were placed in sterile plastic bags and carried to the Plant Pathology Laboratory of Tuscia University for further analyses. To isolate the putative causal fungal agent, twig segments were surface‐sterilized with 3% sodium hypochlorite for 3 min, rinsed twice with sterile distilled water, and dried under a laminar flow. One‐centimeter sections from both healthy and symptomatic tissues were aseptically placed onto Potato Dextrose Agar (PDA) plates and incubated at 25°C ± 1°C for 5–7 days. Fifty pure fungal cultures were obtained through serial transfer of emerging colonies onto fresh PDA plates. Following an initial screening, subsets of isolates were selected for further analyses: (i) 50 isolates for molecular identification based on the *ITS* region, (ii) 10 isolates for a more comprehensive phylogenetic analysis based on the *ITS*, *TUB2*, *CAL*, *TEF‐1*, and *HIS3* regions, and (iii) three isolates for the mycelial extension study across a range of controlled temperatures.

### 2.2 DNA Extraction and PCR Amplification for Molecular Characterization of *Diaporthe Amygdali* Isolates

Mycelium was scraped from the 50 isolates with a sterile scalpel, and genomic DNA was extracted using the Plant Genomic DNA Extraction Mini Kit (Fisher Molecular Biology). Initial molecular characterization of the isolates was carried out by PCR amplification and Sanger sequencing of the ribosomal Internal Transcribed Spacer (*ITS*), as detailed below.

Subsequent molecular analyses were conducted on 10 selected isolates, each originating from different cultivars or from distinct plants within the same cultivar, to represent a broad range of host diversity. Additional amplification and sequencing of the β‐tubulin (*TUB2*), elongation factor (*TEF‐1*), calmodulin (*CAL*), and histone 3 (*HIS3*) gene regions were carried out using the primers reported in Table S1. For each PCR reaction, 40 ng of template DNA was combined with 1× GoTaq Green MasterMix (Promega Corporation), 0.5 μM of both forward and reverse primers, in a final volume of 25 μL. The thermal cycling protocol consisted of an initial denaturation step of 3 min at 94°C, followed by 35 cycles of denaturation at 94°C for 30 s, annealing for 30 s at 48°C for *CAL* gene, 51°C for the *TUB2*, 55°C for the *ITS* and *TEF‐1*, 57°C for the *HIS3*, followed by 30 s of elongation at 72°C and a final extension at 72°C for 5 min. An aliquot of each amplified product was visualized on a 1.2% agarose gel, and the remaining product was sent to Eurofins Genomics for Sanger sequencing.

### 2.3 Phylogenetic Analysis of *Diaporthe* Isolates

The quality of Sanger electropherograms was assessed using FinchTV v.1.4 (available at https://digitalworldbiology.com/FinchTV, accessed on 15 May 2024). For phylogenetic analysis of the *ITS* region, sequences from *Diaporthe* spp. isolates and reference sequences from GenBank were used (Table S2). *Diaporthella corylina* CBS 121124 was included as the outgroup. Sequences were trimmed and concatenated using UGENE v48.1 and subsequently aligned with MUSCLE v3.8.31 (Edgar 2004). A maximum likelihood (ML) phylogenetic tree was built using RAxML‐HPC v8.2.12, with the GTRCATI substitution model and 1000 bootstrap replicates. The trees were visualized using FigTree v1.4.4 (available at http://tree.bio.ed.ac.uk/software/figtree/), and further edited with Inkscape v0.92 (available at https://inkscape.org).

A more comprehensive phylogenetic analysis was carried out through concatenated sequences from five genomic regions. Sequences were concatenated using seqkit v0.16 (Shen et al. 2016) and aligned with *Diaporthe* isolates representing diverse species (Table S2) using MUSCLE. As done for the *ITS* analysis, the resulting alignment file served as input for maximum likelihood (ML) phylogenetic tree construction using RAxML‐HPC v8.2.12 (Stamatakis 2014), with GTRCATI substitution model and 1000 bootstrap replicates. The tree was visualized using FigTree and further edited with Inkscape.

### 2.4 Morphological Identification and Pathogenicity Test

The 50 isolates were categorized based on distinct morphological colony patterns observed on Potato Dextrose Agar (PDA) medium. Representative isolates from each morphotype were selected for further analysis. Three representative isolates, one per morphotype, were selected for further analysis. Then, they were transferred onto PDA, Malt Extract Agar (MEA), and Oatmeal Agar (OA) media, and incubated at 25°C in darkness. After 14 days, the colony's morphology and pigmentation were recorded.

Pathogenicity was assessed by inoculating six one‐year‐old twigs of the ‘Redhaven’ cultivar with the *D. amygdali* isolate DA‐1. Twigs were first washed with sterile distilled water and surface‐sterilized using 3% sodium hypochlorite for 3 min, followed by three rinses with sterile distilled water and drying under laminar airflow. A wound was made at the midsection of each twig using a sterile scalpel and filled with a mycelial disc from a 10‐day‐old *D. amygdali* isolate. The inoculation site was sealed with parafilm, and the twigs were placed in sterile glass tubes containing 3 mL of sterile distilled water, which were then sealed with perforated parafilm. The inoculated twig‐tubes were incubated at 25°C for 14 days, during which symptom development on both the twig surface and subcortical tissue was evaluated after debarking. Sporulation was induced using the same inoculation procedure. After 30 days of incubation at 25°C, the mean length and width of 30 randomly selected conidia were measured under a light microscope (Leitz) at 40× magnification. All experiments were conducted in duplicates to ensure and assess reproducibility.

### 2.5 Experimental Design for Mycelial Extension at Different Constant Temperatures

Based on morphological characterization, the mycelial extension rates of three selected *D. amygdali* isolates were assessed across eight constant temperatures (5°C, 10°C, 15°C, 20°C, 25°C, 30°C, 35°C, and 40°C) to analyze the effect of temperature as a primary growth factor. Four replicates per isolate per temperature accounted for the potential growth variability. To enhance precision and increase the number of repetitions, radial extension was measured in four orthogonal directions (north, west, south, east), following the methodology of Brugneti, Rossini, et al. (2024); Brugneti, Turco, et al. (2024). A 5 mm ∅ diameter mycelial plug, excised from the actively growing margins of 7‐day‐old colonies of each *Diaporthe* isolate, was placed at the center of Potato Dextrose Agar (PDA) plates. Plates were incubated at the above‐mentioned temperatures for the overall duration of the experiment. Mycelial extension was measured using a caliper every 24 h for three consecutive days, ensuring that colonies did not reach plate edges during the measurement period.

### 2.6 Lethal Temperature Threshold for Mycelial Growth

Thermal tolerance was assessed using 5 mm diameter mycelial plugs excised from the actively growing margins of 7‐day‐old colonies of each *Diaporthe* isolate. Plugs were individually placed into 2 mL microcentrifuge tubes and subjected to a temperature gradient from 40°C to 55°C, in 1°C increments, using a temperature‐controlled water bath (Argolab WB 12 L). A 1‐min preheating step was applied prior to a 10‐min heat treatment at each temperature. Four biological replicates were included per isolate. Following heat exposure and subsequent cooling to room temperature, the plugs were transferred, mycelial side down, onto Potato Dextrose Agar (PDA) plates. Plates were incubated at 25°C in darkness for 7 days. Mycelial extension was then recorded to evaluate viability, following the procedure described by Cao and Li (2022).

### 2.7 Data Analysis of Mycelial Extension Rate Over Temperature

The length of the radii measured according to Section 2.3 at different constant temperatures was analyzed as follows. Daily values, expressed in mm day^−1^, were obtained by dividing the measurements taken on the third day by three. After the conversion, two steps were carried out: the script and the dataset to fully reproduce the results of this part of the study are publicly available at https://github.com/lucaros1190/DAMyceliumExtension.

Step 1: Statistical differences, in terms of mycelium extension rate, among the isolates were assessed through a Generalised Linear Model with negative binomial distribution and mixed effect (GLMM), followed by the Bonferroni adjusted as a post hoc test (*α* = 0.05). The assumptions for the application of GLMM were assessed through the R package DHARMa (https://github.com/florianhartig/DHARMa, accessed on 21 June 2025). The isolate and the temperature were considered as independent variables, the replicate as random effect, and the measured radius as dependent variable. Calculations were carried out through the R software using the functions *glmer.nb*() within the package *lme4* (Bates et al. 2015) and the emmeans() and pairs() functions within the R package *multcomp* (Hothorn et al. 2008). Note that for this first step of the analysis differences between temperatures were not taken into account, as the main focus was to identify the fastest isolates.

Step 2: Mycelium extension over temperature for each isolate was quantified by fitting the data with the Briére equation (Briere et al. 1999):

(1) $$R\left(T\right)=\mathrm{aT}\left(T-T_{L}\right)$$

where T is the temperature of growth, $T_{L}$ and $T_{M}$ are the minimum and the maximum temperature below and above which mycelium extension is theoretically not possible, respectively, and a and m are empirical parameters with no biological meaning. The best fit parameters and their standard errors were obtained by a non‐linear least square regression, carried out through an ad hoc Python (v. 3.9) script that involved the numpy and scipy packages. The goodness of fit, instead, was evaluated considering the coefficient of determination $R^{2}$ (Bellocchi et al. 2011; Ikemoto and Kiritani 2019; Rossini et al. 2020, 2021) Additional information coming from Equation (1), and reported as a result, is the optimal temperature for the development, corresponding to its maximum.

## 3 Results

### 3.1 Field Survey and Fungal Isolation

**FIGURE 1:**
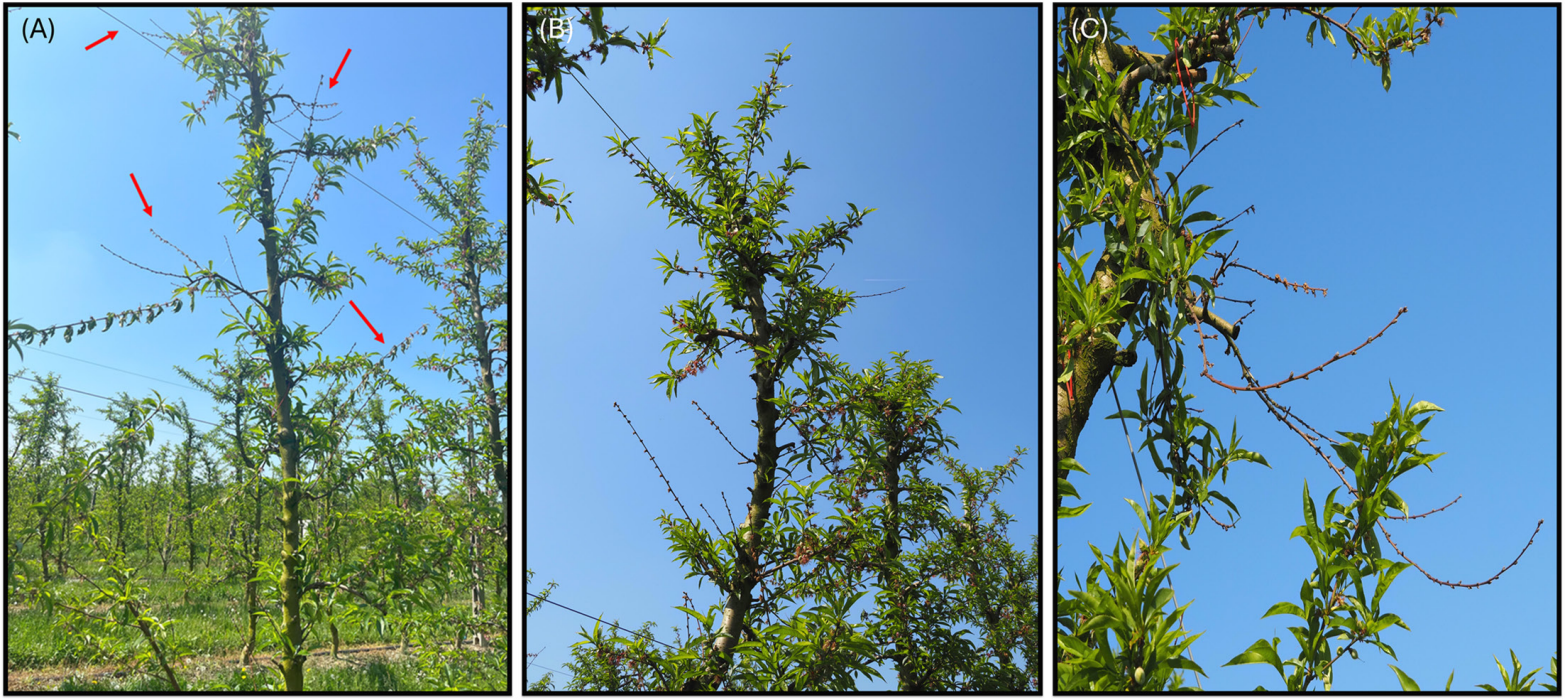
Blighted shoots (red arrows) among the green canopy of peach trees of the varieties “Pavoro—Pav 2708” (A), “Pavoro—Pav 1705” (B), and “Lami.COM” (C).

**FIGURE 2:**
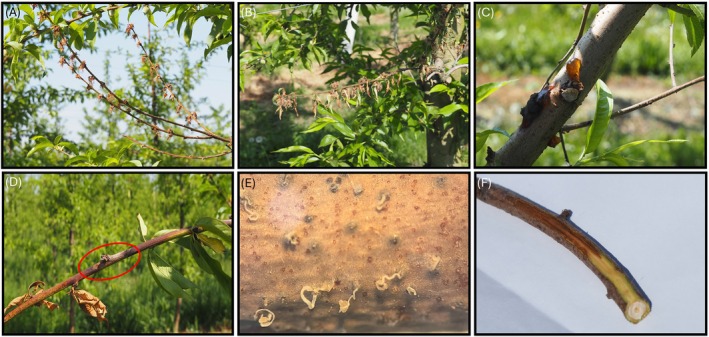
Symptoms observed on peach twigs infected by *Diaporthe amygdali*: Dry leaves and flowers (A, B); Gummosis in the canker area (C); Characteristic cankers along the shoots, brown or silvery‐grey in colour (red ring) sunken in correspondence with the buds (D); Black pycnidia with the presence of abundant formation of conidia in creamy yellow masses (E); Browning under the crust of the symptomatic areas (F).

Peach trees of different cultivars involved in this study showed a high incidence of twig blight, shoot dieback, and canker symptoms. Several blighted shoots interspersed within the green canopy (Figure 1A–C) were detected through visual inspections of the trees. Infected twigs and shoots showed desiccated leaves and flowers (Figure 2A,B), with occasional gummosis observed at canker sites (Figure 2C). Distinctive cankers, characterized by brown or silver‐gray discoloration, were present along the shoots, particularly at bud locations (Figure 2D). Black pycnidia were visible on the surface of the gray necrotic tissue, together with abundant conidia in yellow‐cream masses (Figure 2E). Subcortical tissue in symptomatic areas showed extensive browning (Figure 2F).

### 3.2 Molecular Identification of *D. amygdali*

**FIGURE 3:**
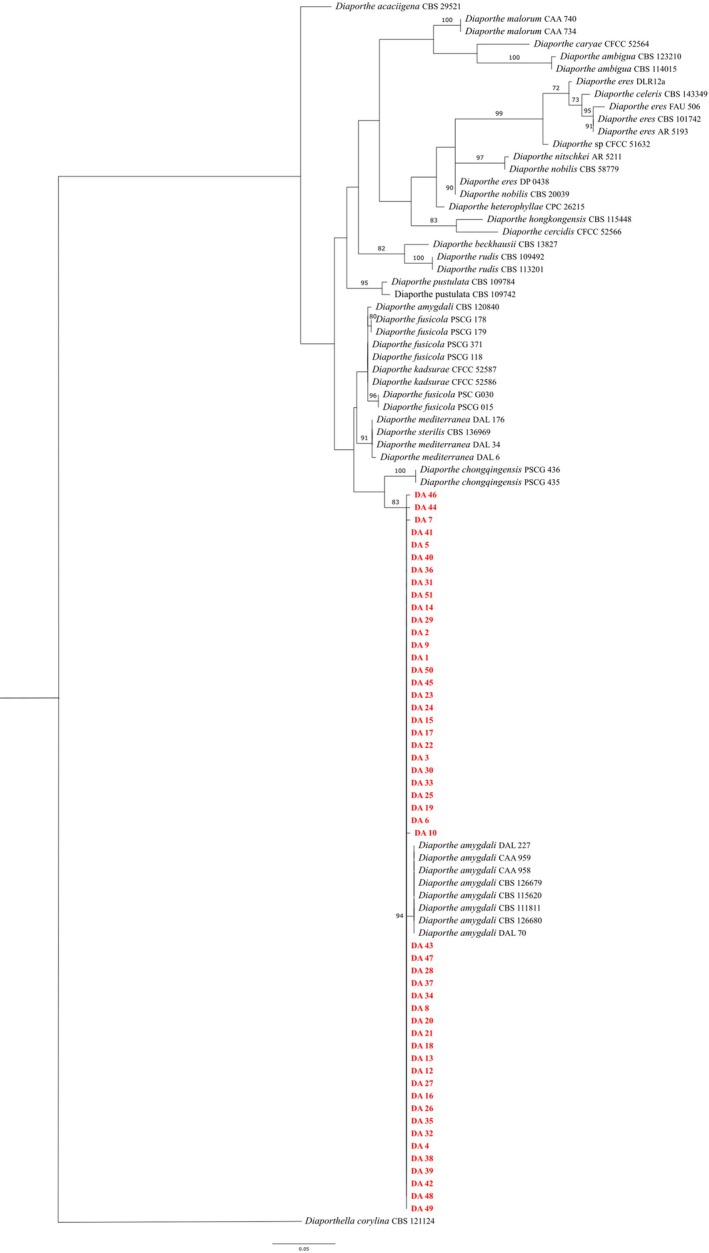
Maximum likelihood (ML) phylogenetic tree of the concatenated sequences of *ITS* among isolates of the *Diaporthe* spp. The tree was rooted on the *Diaporthella corylina*. Bootstraps values indicating the robustness of the clustering are reported as node values.

**FIGURE 4:**
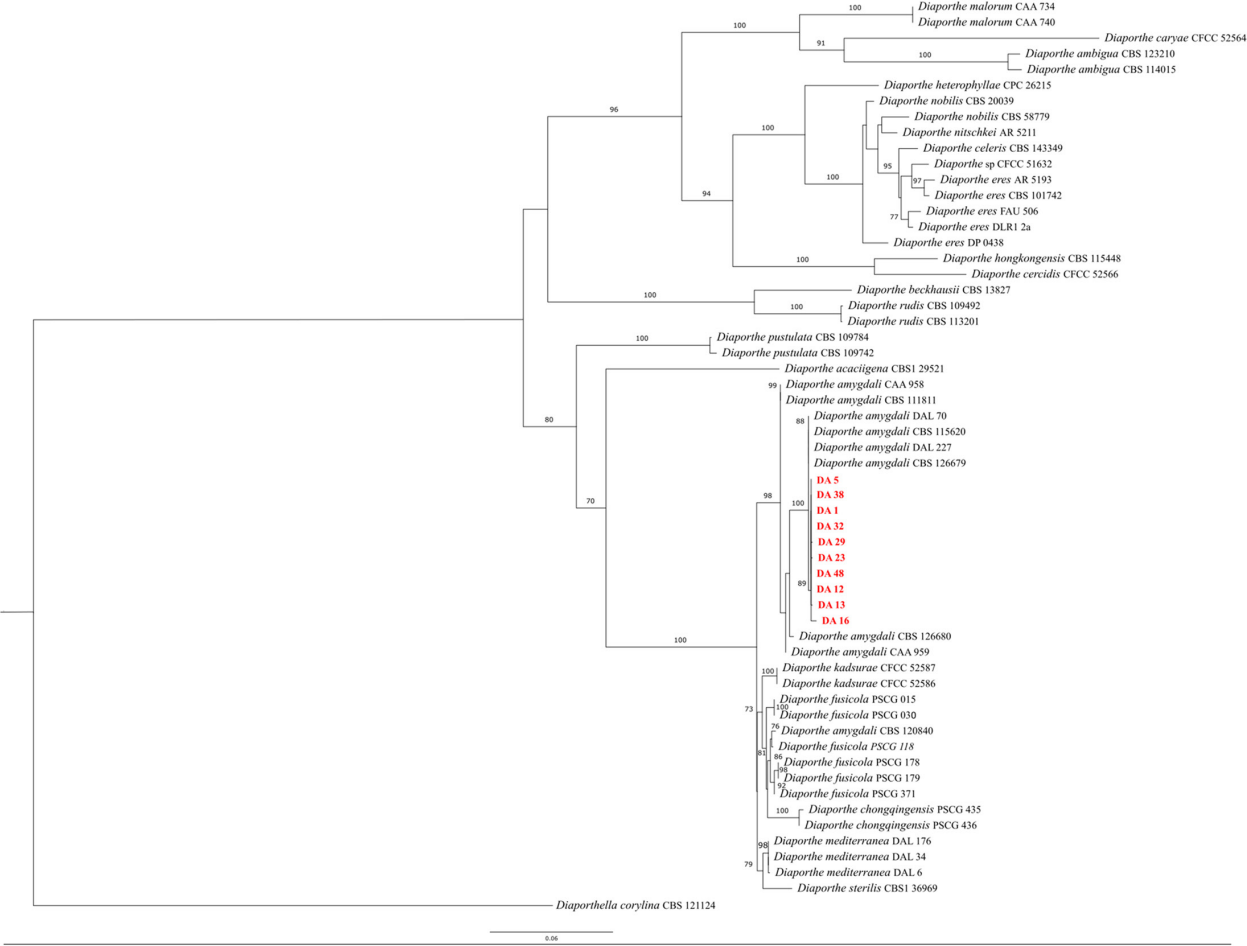
Maximum likelihood (ML) phylogenetic tree of the concatenated sequences of five of housekeeping genes among isolates of the *Diaporthe* spp. The tree was rooted on the *Diaporthella corylina*. Bootstraps values indicating the robustness of the clustering are reported as node values.

**FIGURE 5:**
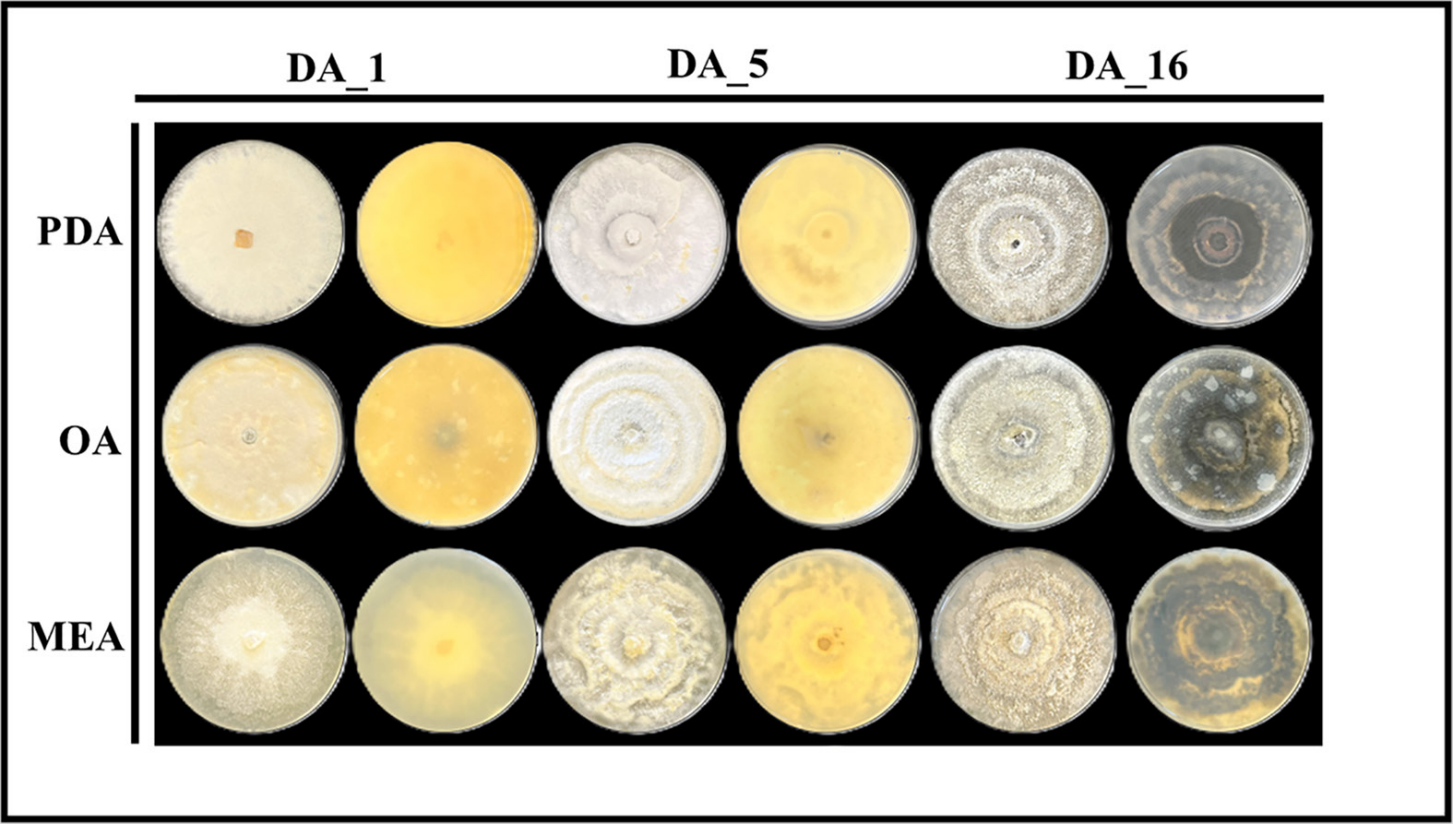
Morphological features of *Diaporthe amygdali* isolates DA‐1, DA‐5, and DA‐16, corresponding to morphotypes one, two, and three, respectively, grown on PDA, OA, and MEA after 14 days at 25°C. Isolate DA‐1 (morphotype one) shows flat, compact ivory mycelium on PDA and OA, with loose margins and a white center on MEA. Isolate DA‐5 (morphotype two) forms fluffy white mycelium with irregular rings and ivory spots on PDA and OA, and loose, dirty white‐ivory rings on MEA. Isolate DA‐16 (morphotype three) exhibits flat grey to olivaceous grey mycelium with irregular rings, darker undersides on PDA and OA, and cream‐colored shading on MEA.

Overall, 50 isolates were recovered from 55 symptomatic peach twigs and initially identified as *Diaporthe* spp., according to morphological characteristics (Figure 5). BLASTn analysis of the 50 *ITS* sequences with the GenBank database confirmed that all isolates belonged to *D. amygdali*. This molecular classification was further supported either by *ITS* or multi‐locus phylogenetic analyses (Figures 3 and 4), which confirmed clustering of the isolates within the *D. amygdali* clade. Sanger sequences and associated data were deposited in the NCBI GenBank database (Table S3).

### 3.3 Morphological Characterization and Pathogenicity Test of *D. Amygdali*

#### 3.3.1 Morphological Characterization of *D. Amygdali* Isolate

According to the morphological traits of the 50 isolates grown on PDA medium, we obtained three different morphotypes: the isolates DA‐1, DA‐5, and DA‐16 represent morphotypes one, two, and three, respectively (Figure 5).

Culture characteristics of morphotype one: on PDA at 25°C after 14 days, the colony was characterized by a flat compact mycelium, ivory‐colored on the surface and cream‐colored on the underside. On OA medium at 25°C after 14 days, the mycelium was compact and ivory‐colored on both the surface and the bottom of the plate. On MEA medium at 25°C after 14 days, the mycelium was loose at the colony's margin and compact with a white color at the center.

Culture characteristics of morphotype two: on PDA at 25°C after 14 days, the colony was characterized by flat, white, and fluffy mycelium, which spread to form irregular rings, dense with solid ivory‐colored spots. The bottom was cream‐colored with brownish spots in the central region. On OA medium at 25°C after 14 days, the colony was characterized by white fluffy mycelium, flat and forming irregular rings with ivory‐colored margins. On MEA medium at 25°C after 14 days, the aerial mycelium was loose and spread into irregular rings of a dirty white‐ivory color, with the bottom of the plate cream‐colored.

Culture characteristics of morphotype three: on PDA at 25°C after 14 days, the colony was characterized by flat gray mycelium, which spread to form irregular rings, dark gray‐black on the underside of the plate. On OA medium at 25°C after 14 days, the colony consisted of flat, olivaceous gray, fluffy mycelium, which expanded to form irregular rings with an olivaceous gray bottom. On MEA medium at 25°C after 14 days, the colony was characterized by flat, olivaceous gray mycelium, with an olivaceous gray surface and cream‐colored shades on the bottom.

**FIGURE 6:**
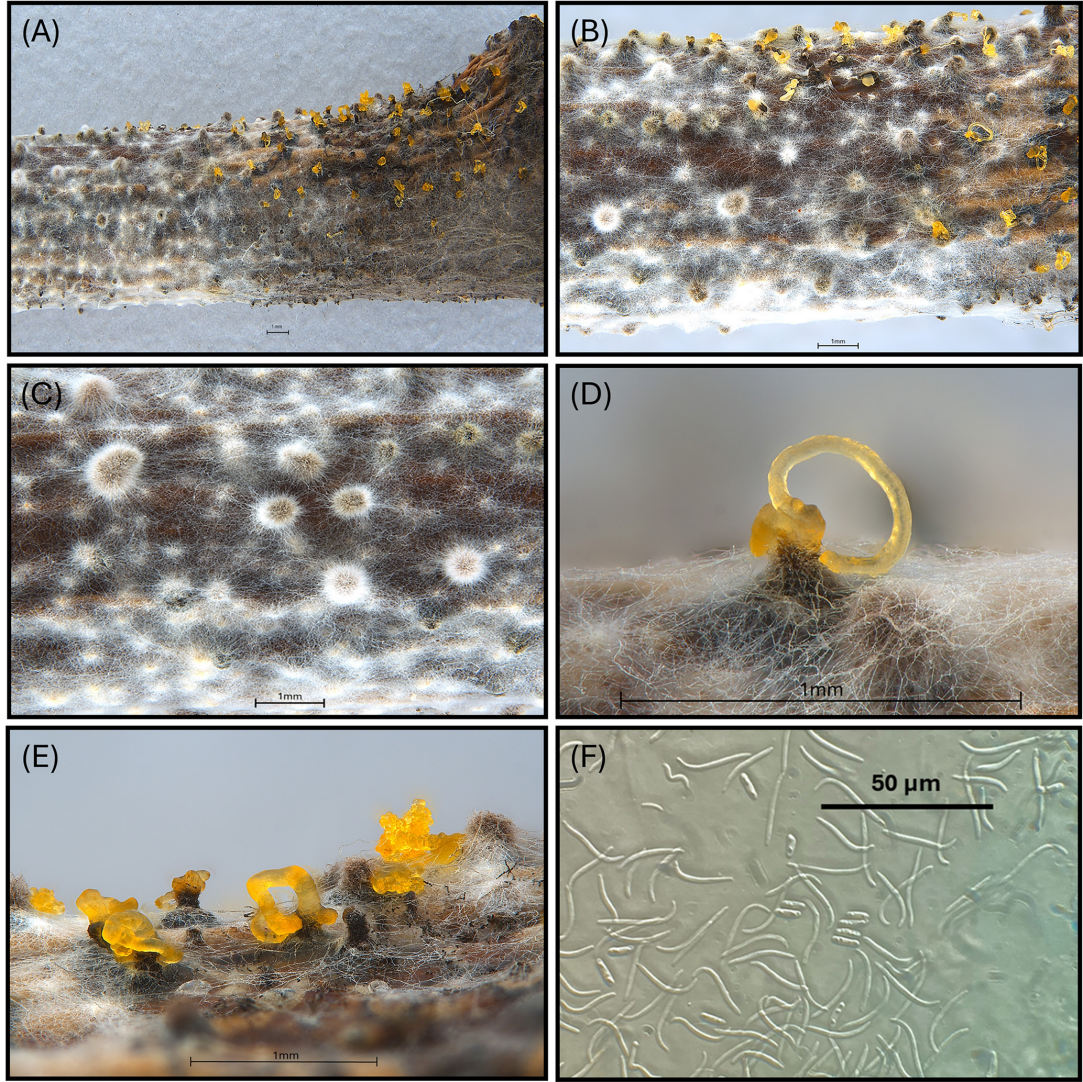
Fructification of *Diaporthe amygdali* isolate DA‐1 on one‐year‐old peach twigs (cv. ‘Redhaven’) 30 days after artificial inoculation. Black pycnidia and conidial cirrus formations; α‐conidia (fusiform conidia) and β‐conidia (filiform conidia). (A) Overview of pycnidial fructifications and cirrus formation on the inoculated twig surface. (B) Close‐up view showing pycnidia at various stages of maturation, including both closed pycnidia and those exuding mucilaginous conidial cirri. (C) Detail of immature, closed pycnidia without visible cirrus production. (D) Detail of a mature pycnidium with mucilaginous cirrus emerging through the ostiole. (E) Group of mature pycnidia actively releasing mucilaginous conidial cirri. (F) Microscopic view of conidia: α‐conidia are fusiform, hyaline, and aseptate; β‐conidia are filiform, curved, and hyaline.

The pycnidial conidiomata on the peach twigs after 30 days of incubation at 25°C were dark and globose, solitary or aggregated, erumpent on the surface of the twig, bordered by white hyphae with dimensions between 132.5 and 503.5 μm. When mature, yellow cirri erupted from the pycnidia surface. α‐conidia appeared fusiform, hyaline, aseptate, monoguttulate, but in most cases biguttulate, with dimensions 5.5–8 × 1.5–3.0 μm, while the β‐conidia were one‐celled, hyaline, and filiform with 17–30 × 1–1.5 μm (Figure 6).

#### 3.3.2 Pathogenicity Test of *D. amygdali* on Peach Twigs

**FIGURE 7:**
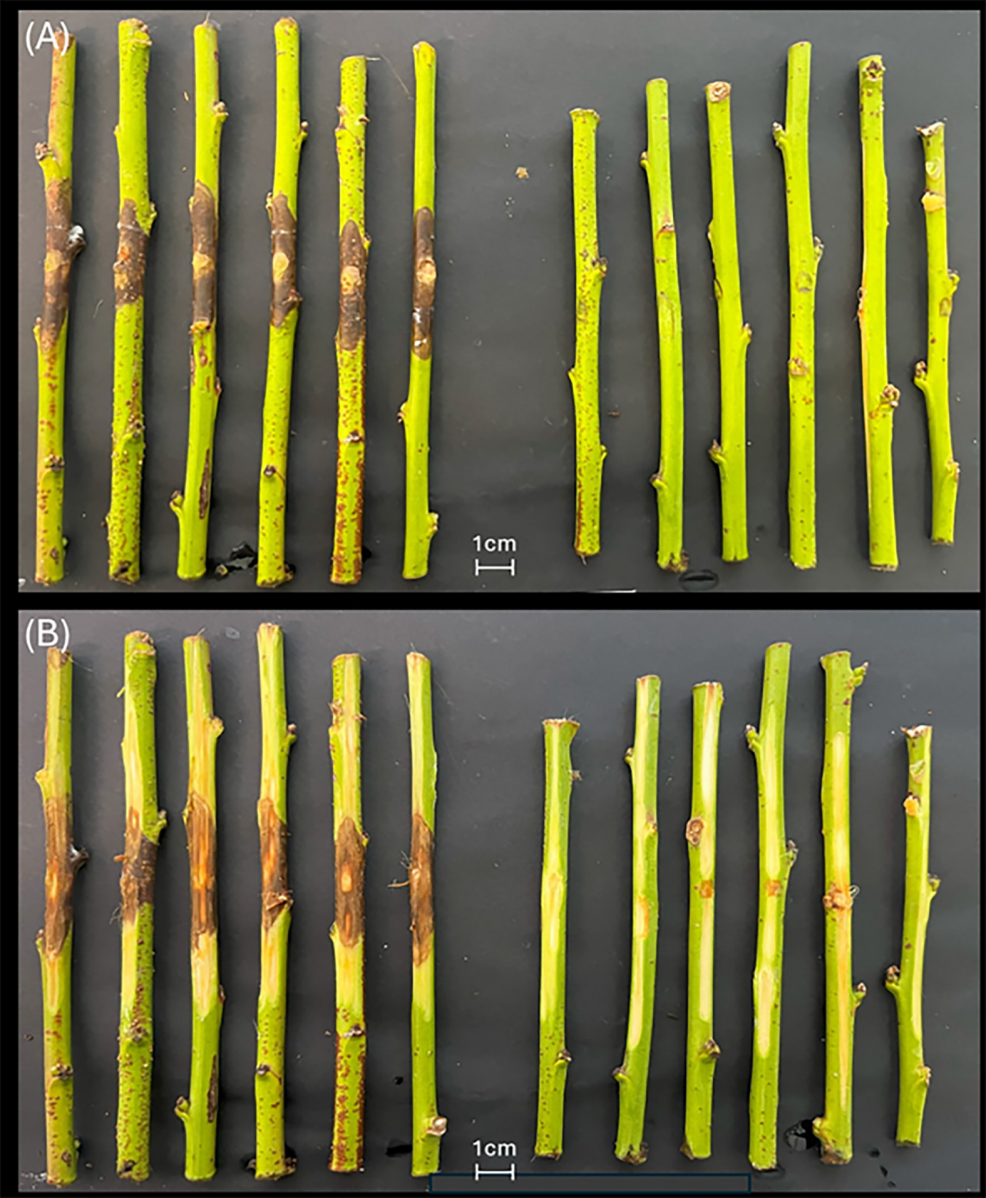
Necrotic lesions on ‘Redhaven’ cultivar twigs after inoculation with a mycelial disc of the *D. amygdal*i DA‐1isolate and incubation at 25°C for 14 days; In panel (A), inoculated twigs are shown on the left, while control twigs are on the right. In panel (B), the same twigs are shown after bark removal to reveal the lesions: Treated twigs are on the left, and controls on the right.

Pathogenicity tests were conducted on six one‐year‐old twigs of the ‘Redhaven’ cultivar using the *D. amygdali* DA‐1 isolate. Fourteen days post‐inoculation, all twigs showed necrotic lesions on the surface, with the necrosis extending into the subcortical tissue (Figure 7 A‐B), confirming the symptoms observed in the field (Figure 2). Control twigs remained asymptomatic. Furthermore, the morphology of the fungal colonies re‐isolated from the symptomatic twigs closely matched the original inoculum, fulfilling Koch's postulates.

### 3.4 Mycelial Extension at Different Constant Temperatures

**FIGURE 8:**
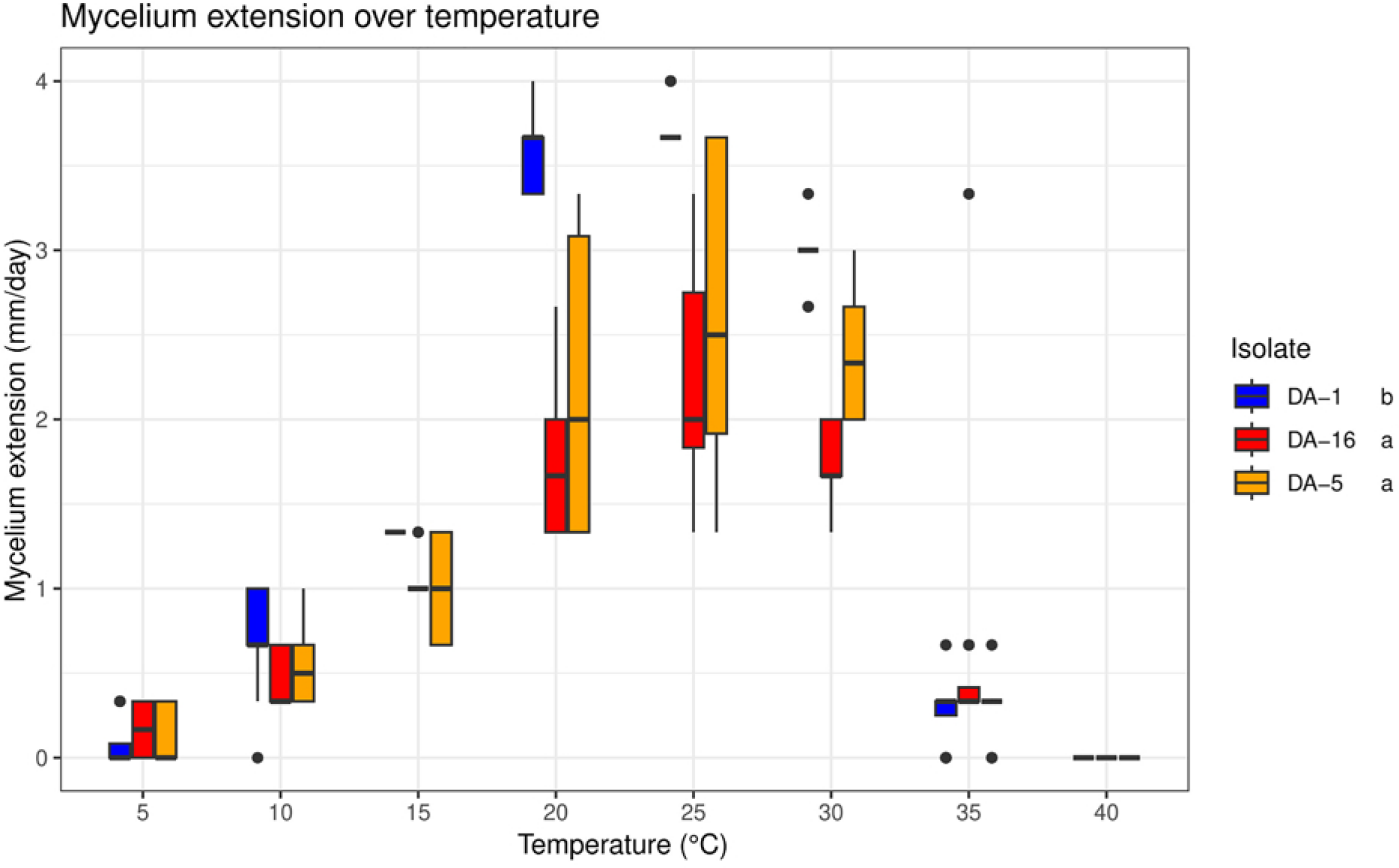
Mycelium extension at different constant temperatures. Lines inside the boxes indicate the median values and the points outside represent the outliers. The whiskers include 95% of the data. The different letters reported in the legend indicate significant difference between the isolates after Bonferroni *post hoc* (*p* < 0.05).

Proceeding by order, the first step of the analysis showed statistical differences, in terms of mycelium extension rate, between the three isolates considered for this part of the study: DA‐1, DA‐5, and DA‐16. The isolate DA‐1 showed a higher mycelium extension rate (Figure 8), being significantly different from the isolate DA‐5 (GLMM, *Z* = 3.46, *p* = 0.002, NDF = Inf) and DA‐16 (GLM, *Z* = 2.609, *p* = 0.03, NDF = Inf). No differences, instead, were observed between the isolates DA‐5 and DA‐16 (GLM, *Z* = −0.88, *p* = 1, NDF = Inf).

**FIGURE 9:**
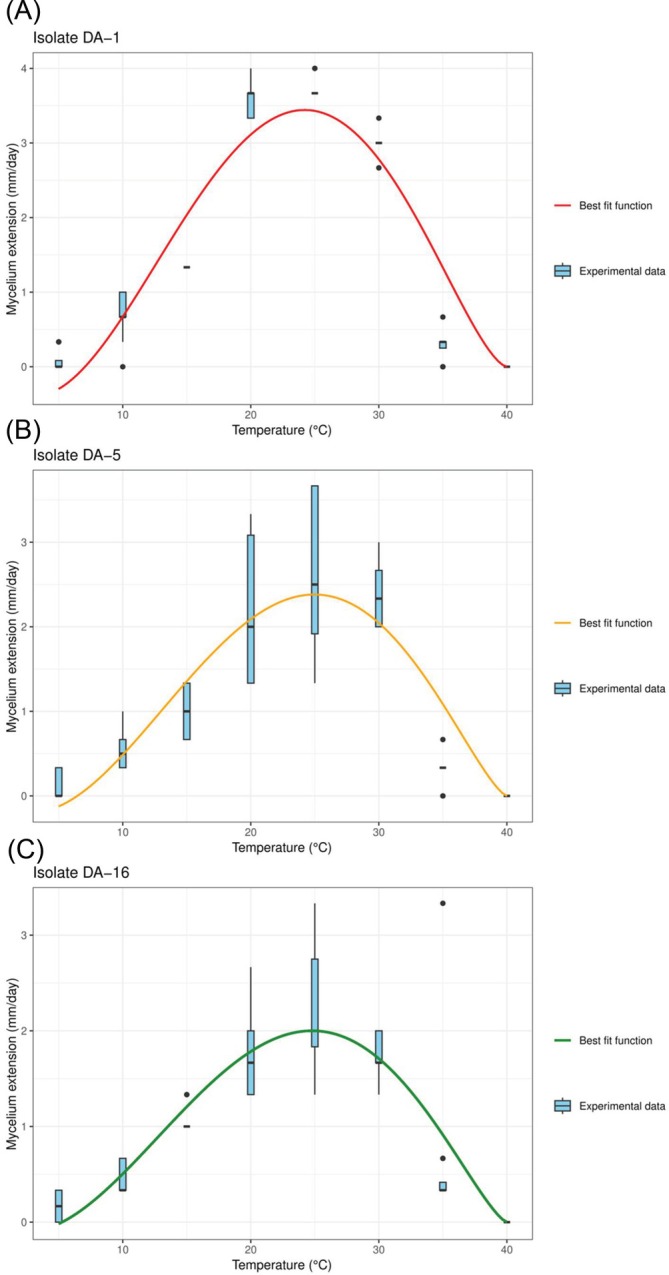
Mycelium extension rate, isolates DA‐1, DA‐5, and DA‐16. Comparison between the experimental dataset (box plot) and the best fit Equation (1). Lines inside the boxes indicate the median values and the points outside represent the outliers. The whiskers include 95% of the data. The best fit parameters corresponding to the three best fit equations are listed in Table 1.

The statistical differences observed in the first step of the analysis were further confirmed by the best fit parameters estimated by fitting the datasets with Equation (1). From the values listed in Table 1 it is possible to observe that the main difference between the isolates is in terms of minimum temperature threshold. The maximum temperature threshold, in fact, is 40°C for the overall isolates, while the minimum temperature for the mycelium extension was 5°C for DA‐16, 6°C for DA‐5, and 7°C for DA‐1. The values of the optimal temperatures were mostly comparable as well, and centered around 24°C. Plots that compare the best fit functions with the corresponding raw datasets are reported in Figure 9.

**TABLE 1:** Best fit parameters of the Equation (1) for the three *Diaporthe amygdali* isolates DA‐1, DA‐5, and DA‐16.

Isolate	Brière function's parameters	Goodness of fit and optimal temperature
DA‐1	*a* = (1 ± 1)·10^−4^	*R* ^2^ = 0.872
	*T*_L_ = 7.0 ± 0.6	NDF = 92
	*T*_M_ = 40 ± 2	*T*_opt_ = 23 ± 1
	*m* = 0.6 ± 0.1	
DA‐5	*A* = (1 ± 1) × 10^−4^	*R* ^2^ = 0.732
	*T*_L_ = 6 ± 1	NDF = 92
	*T*_M_ = 40 ± 3	*T*_opt_ = 24 ± 1
	*M* = 0.7 ± 0.2	
DA‐16	*a* = (1 ± 1)·10^−4^	*R* ^2^ = 0.736
	*T*_L_ = 5 ± 1	NDF = 92
	*T*_M_ = 40 ± 3	*T*_opt_ = 24 ± 1
	*m* = 0.7 ± 0.2	

*Note:* The goodness of fit is expressed by the coefficient of determination $R^{2}$, while NDF indicates the number of degrees of freedom and $T_{\mathrm{opt}}$ the optimal temperature. A graphical representation of the best fig functions is shown in Figure 9.

## 4 Lethal Temperature Threshold for Mycelial Growth

Ressults highlighted different thermal tolerances among the three isolates analyzed (DA‐1, DA‐5, and DA‐16). After a 10‐min heat treatment, the three isolates showed mycelial growth at temperatures ranging from 40°C to 51°C, indicating a shared baseline heat resistance. However, significant differences emerged at higher temperatures. Notably, isolate DA‐5 was still able to grow up to 53°C, while isolate DA‐16 demonstrated an even greater thermal tolerance, with growth observed at 54°C. Conversely, DA‐1 did not grow beyond 51°C. None of the isolates showed mycelial growth after exposure to 55°C, suggesting that this temperature represents a critical threshold beyond which the mycelial structures are irreversibly compromised (Table S4).

## 5 Discussion and Conclusions

The genus *Diaporthe* encompasses a complex and ecologically diverse group of fungi which can have different roles: plant pathogens, endophytes, and saprobes. The historical classifications of *Diaporthe* species were largely based on host specificity, which have led to an incomplete understanding of their actual host ranges. However, contemporary phylogenetic and multi‐locus studies have shown that many taxa are active on broad host ranges and have significant capabilities for inter‐host migration (Yang et al. 2018; Arciuolo et al. 2021; Dissanayake et al. 2024). The detrimental impact of *Diaporthe* species on *Prunus* plants, encompassing peach, almond, and cherry, is well documented in recent scientific literature. Wang et al. (2021) identified seven *Diaporthe* species associated with canker and dieback on peach trees in China, revealing both previously known ( *D. caryae* , *D. cercidis*, *D. eres*, *D. hongkongensis*, *D. unshiuensis*) and newly identified pathogens (*D. jinxiu* and *D. zaofenghuang*). This research was further expanded by Zhou et al. (2024) who observed the association of *D. caulivora* and *D. discoidispora* with peach twig canker, highlighting the ongoing discovery of pathogenic species. Chen et al. (2023) also contributed to this understanding by identifying *D. eres* and *D. hongkongensis* as being associated with Cherry Trunk Diseases in China, emphasizing the pathogen's effect on multiple *Prunus* species. *D. amygdali*, first documented in 1936, has been extensively studied as a primary pathogen of almond trees, particularly in Spain (León et al. 2020; Beluzán et al. 2025).

While the presence of *D. amygdali* in Italy is well‐established concerning almond trees (Gusella et al. 2023), its role as a pathogen of peach trees has received less scientific attention, despite being the primary causal agent of twig canker and branch symptoms (TCBS), particularly in the Emilia‐Romagna region (Regione Emilia Romagna‐Agricoltura 2008; Agronotizie 2021).

In our survey, symptoms observed during field surveys were consistent with observations carried out on peaches in other areas worldwide and attributed to the *Diaporthe* genus. Molecular analyses, based on the *ITS* region and four additional loci, closely clustered the isolates within the *D. amygdali* clade, which now includes species such as *D. mediterranea* , *D. garethjonesii*, *D. sterilis* , *D. kadsurae*, *D. ternstroemia*, *D. ovoicicola*, *D. fusicola*, and *D. chongqingensis* (Hilário et al. 2021; Dissanayake et al. 2024). The results obtained in this study did not show significant differences with respect to previous morphological characterizations of *D. amygdali* isolates. Previous studies consistently described the absence of β‐conidia in isolates of various hosts (Delacroix 1905; Mostert et al. 2001; León et al. 2020; Hilário et al. 2021, Beluzán et al. 2021; Gusella et al. 2023) or defined them as rare and sporadic (Tuset and Portilla 1989). However, our results showed the presence of β‐conidia in peach isolates following artificial inoculation of ‘Redhaven’ twigs. These β‐conidia exhibited morphological features and dimensions comparable to those previously observed on papaya and walnut (Meng et al. 2018; Alam et al. 2023) but longer and less wide than those observed by Tuset and Portilla (1989). This novel observation suggests that β‐conidia production may be host‐ or condition‐dependent and warrants further investigation into the environmental or physiological factors influencing their development. Field observations carried out in this study have provided valuable insights on the climate change effect on fungal reproduction, dispersal, and survival. Notably, pycnidial fruiting and the emergence of cirrus were observed as early as April, a considerable shift with respect to the time reported by previous studies. The difference is of almost 3 months, as fruiting typically occurs in the summer according to the literature (Tuset and Portilla 1989; Adaskaveg 2002).

This information can further support monitoring, as there is a more precise indication of when early detection surveys should be carried out.

Mycelial extension rates under different constant temperatures, and the mathematical interpolation of the results is additional relevant and new information that this study provided. The isolates showed slightly different extension rates, suggesting some slight ecophysiological differences that might affect the appearance and the spread of the disease. According to this part of the results, moreover, the isolate DA‐1 has a faster mycelium extension than the others, but at the same time the three of them showed optimal temperatures between 23°C and 24°C, 1°C and 2°C lower than the 25°C observed by Mostert et al. (2001), Hilário et al. (2021), and Gusella et al. (2023). Moreover, the capability of the mycelium to survive under extreme environmental conditions that exceed 51°C, even if for a limited amount of time, may offer the fungus more chances to survive during the summer period, even in the case of plants fully exposed to the sun.

In conclusion, the results of this study provide novel insights on *D. amygdali* on peach trees in Italy, particularly in the Emilia‐Romagna region, laying the foundations for more in‐depth studies. The morphological and molecular characterization of the main fungal structures and of the reproductive organs, as well as the pathogenicity test on peach cultivars is a starting point to better understand either plant susceptibility or the different isolates involved in TCSB epidemics. The identification of previously unreported characteristics, along with the confirmation of *D. amygdali* as the predominant species, underscores the need for continued surveillance and research to better understand the evolving threats posed by this pathogen in peach production systems. On the other hand, the quantitative information on mycelium extension over temperature is the basis for the development of ad hoc models that might be included in decision support systems.

## Author Contributions

F.B. carried out all the experiments and wrote the original draft of the manuscript. L.R. conceived the mycelial extension experiments, analyzed the data, and revised the manuscript. A.N. helped in the experiments regarding the morphological and molecular characterization of the fungal isolates. M.C. and M.O. provided access to the samples and supervised the study. A.M. conceived and supervised all the experiments. S.T. conceived the experiments, helped with the molecular characterization of the fungal isolates, provided project and funding acquisition, supervised the study, and wrote the manuscript.

## Conflicts of Interest

The authors declare no conflicts of interest.

## Supporting information


**Table S1:** List and sequence of the primers used for the molecular characterization.

**Table S2:** Strains used for the phylogenetic analyses of *Diaporthe* spp. with details about host, location, and GenBank accession numbers.

**Table S3:** List of selected *Diaporthe amygdali* isolates and GenBank accession numbers.

**Table S4:** Lethal temperature for mycelium growth.

## Data Availability

The nucleotide sequences related to this work have been deposited and are available on the NCBI GenBank database under the Accession Numbers: PV453169‐PV453218 (*ITS*); PV476914‐PV476923 (*TUB2*); PV476924‐PV476933 (*HIS3*); PV476934‐PV476943 (*CAL*); PV476944‐PV476952 (*TEF‐1*).
